# Comparison of postoperative analgesia use between robotic and laparoscopic total hysterectomy: a retrospective cohort study

**DOI:** 10.1007/s11701-023-01581-3

**Published:** 2023-03-23

**Authors:** Shohei Tanabe, Ryohei Yamamoto, Sachiyo Sugino, Kotaro Ichida, Kiyoshi Niiya, Syuji Morishima

**Affiliations:** 1 Kobe City Medical Center, West Hospital, 2-4 Ichibancho, Nagata-ku, Kobe, Hyogo 653-0013 Japan; 2 Department of Healthcare Epidemiology, School of Public Health in the Graduate School of Medicine, Kyoto University, Yoshida Honmachi, Sakyo-ku, Kyoto, 606-8501 Japan

**Keywords:** Body mass index, Logistic models, NSAIDs, Postoperative pain

## Abstract

Although robotic and laparoscopic total hysterectomies are widely used as minimally invasive procedures, consensus on which is superior regarding lesser postoperative pain is lacking. This study determines whether there is a difference in the proportion of postoperative use of non-steroidal anti-inflammatory drugs (NSAIDs) and acetaminophen between robotic and laparoscopic total hysterectomies. This retrospective cohort study enrolled patients who underwent robotic or laparoscopic total hysterectomy for uterine fibroids, adenomyosis, or cervical intraepithelial neoplasia grade 3 at a hospital between July 2016 and November 2021. The outcome was postoperative analgesics (i.e., NSAIDs or acetaminophen) use. Unadjusted and adjusted logistic regression analyses were performed to evaluate the association between the procedure and outcome. Adjusted variables were age, body mass index, surgeon’s laparoscopic technique certification, intravenous patient-controlled analgesia, and wound local anesthesia. Of 127 patients, 3 were excluded, and 124 were included. Robotic and laparoscopic hysterectomy was performed in 38 and 86 patients, respectively. Postoperative analgesics were administered to 10 (26.3%) and 52 (60.5%) patients in the robotic and laparoscopic groups, respectively. Unadjusted logistic regression analysis showed significantly more frequent analgesics use in the laparoscopy group (odds ratio [OR] 4.28; 95% confidence interval [CI] 1.85–9.93; *p* < 0.01). Adjusted logistic regression analysis did not detect significant differences (OR 2.62; 95% CI 0.91–7.56; *p* = 0.07). No significant difference in the proportion of postoperative analgesia was observed between robotic total hysterectomy and laparoscopy. Future studies must include larger sample sizes and aligned intraoperative and postoperative analgesic management.

## Introduction

Laparoscopic and robotic minimally invasive total hysterectomies are widely used in gynecological surgery. A comparison of laparoscopic and robotic total hysterectomies reported no difference in operative time, blood loss, or intraoperative complications [1]. Therefore, the outcomes related to postoperative patient-centered outcomes need to be evaluated.

Several studies have been conducted to determine whether robotic or laparoscopic surgery is superior in terms of postoperative pain; however, no conclusions have been reached. In a study that compared both techniques, with postoperative pain scores as the outcome, no significant differences were detected [2]. Likewise, no differences were detected in a similar study in which postoperative opioid consumption was the outcome [3]. However, these studies focused on opioids and did not consider non-opioid use. Guidelines recommend multimodal postoperative analgesic management with non-opioid agents (non-steroidal anti-inflammatory drugs [NSAIDs] and acetaminophen) and local anesthetics in addition to opioids [4]. In other words, to understand whether robotic or laparoscopic surgery is superior for postoperative pain, an outcome assessment that considers non-opioid medications is needed. However, few studies have compared the use of NSAIDs or acetaminophen as outcomes in managing postoperative pain in patients who underwent robotic versus laparoscopic total hysterectomy [5].

This study compares postoperative analgesics use, defined as the use of NSAIDs or acetaminophen, in robotic versus laparoscopic total hysterectomy; we hypothesize that laparoscopic total hysterectomy would cause less postoperative pain than robotic total hysterectomy.

## Methods

### Study design and setting

This was a single-center, retrospective cohort study conducted at a hospital in Kobe, Japan. The hospital has 358 beds, of which 28 are used for obstetrics and gynecology. Annually, approximately 280 surgeries are performed in the obstetrics and gynecology department. The study period was from July 2016 to November 2021. Cases were registered using consecutive sampling, and data were extracted from the medical records. This study was approved by the ethics committee of Kobe City Medical Center, West Hospital (No. 21-040).

### Participants

Patients who underwent robotic or laparoscopic total hysterectomy for uterine fibroids, adenomyosis uteri, or cervical intraepithelial neoplasia grade 3 at our hospital were included in the study. We excluded patients who underwent robotic or laparoscopic surgery and were then converted to open surgery, had an epidural catheter placed preoperatively, or used pentazocine for postoperative analgesia.

### Exposure

**Fig. 1:**
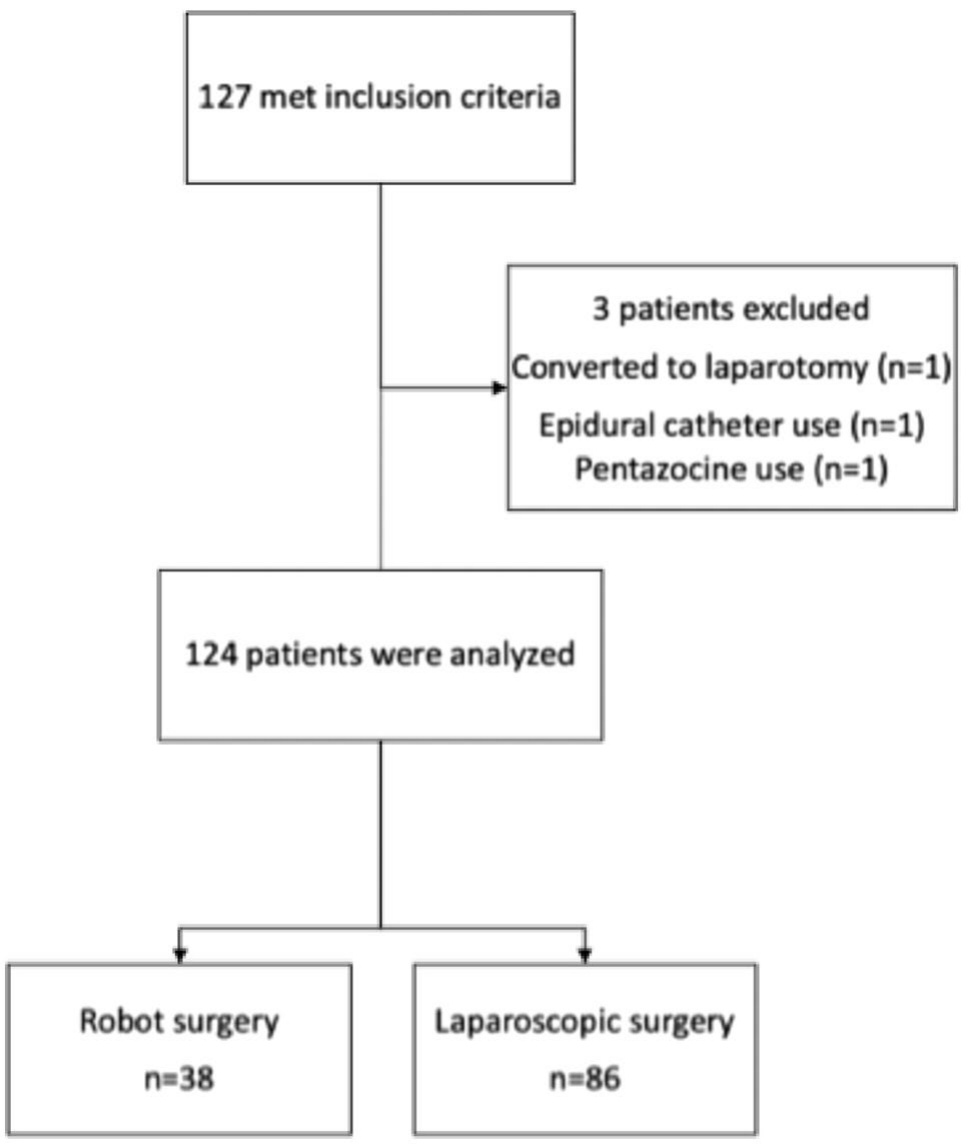
Flow diagram

**Fig. 2:**
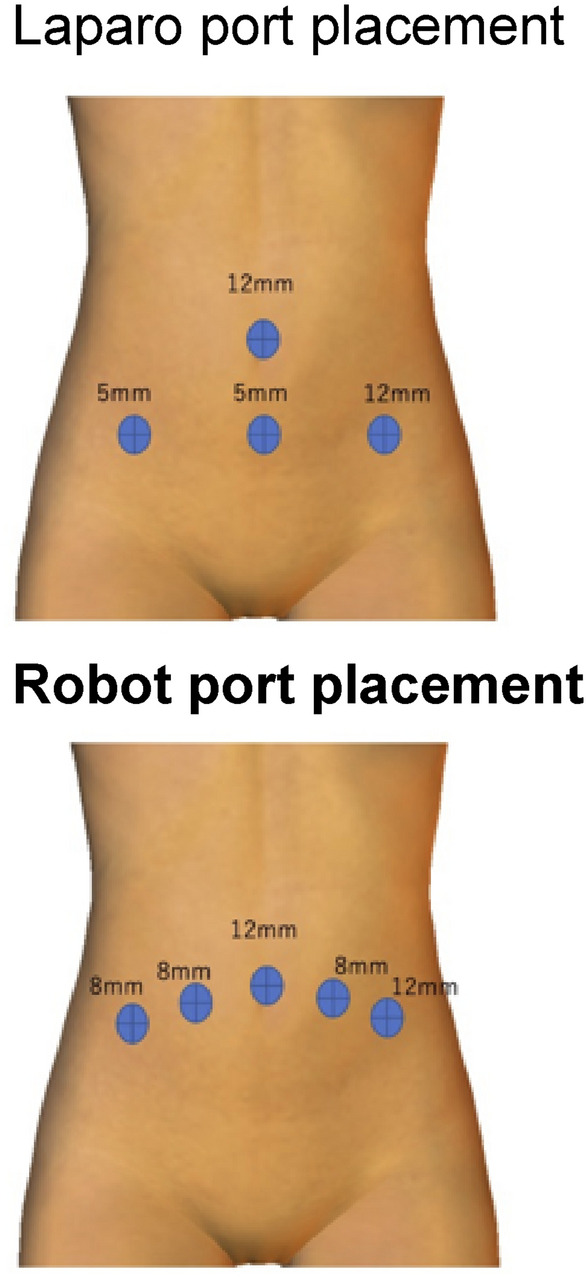
Port placement and diameter for laparo and robot

The primary exposure was laparoscopic total hysterectomy. There were 12-mm ports in the umbilicus and left lower abdomen and 5-mm ports in the mid-lower abdomen and right lower abdomen (Fig. 1). There were no criteria for choosing robotic or laparoscopic technique, and each surgeon made their own subjective decisions. The control procedure was a robotic total hysterectomy. Da Vinci Si (Intuitive Surgical, Sunnyvale, CA, USA) was the equipment used. The ports were 12 mm in the umbilicus and left lower abdomen and 5 mm in two locations in the right lower abdomen and one location in the left lower abdomen (Fig. 2).

### Common management to both groups

General anesthesia was administered intraoperatively to all patients. There were no criteria on whether maintenance anesthesia should be intravenous or inhaled and whether postoperative pain management should involve local anesthesia at the wound site or intravenous patient-controlled analgesia (IVPCA). These were decided by the anesthesiologist. All patients received NSAIDs or acetaminophen within 1 h of surgery completion.

### Outcome

The primary outcome measure was analgesic use (NSAIDs or acetaminophen) from immediately after surgery to 9:00 AM the next day. A charge nurse administered medications if the patient complained of pain; whether to administer NSAIDs or acetaminophen was based on the drug not administered before the end of surgery. There were no explicit rules regarding the degree of pain for which analgesic medications were administered, and the charge nurse subjectively judged the need for analgesics in each case.

### Other measurements

The variables were age, body mass index (BMI), laparoscopic technique certification, IVPCA, and local anesthesia of the wound. Laparoscopic technique certification is a qualification issued by the Japanese Society of Obstetrics and Gynecology Endoscopy to physicians certified by surgical videos as having attained a certain skill level. To ensure the reliability of the variables, we checked the medical and anesthesia records multiple times.

### Statistical analysis

Patient characteristics were described using median and interquartile range (IQR) for continuous variables and number and percentage (%) for categorical variables. Methods of analysis included unadjusted and adjusted logistic regression analyses to examine the association between the operative technique and analgesic use. Adjusting factors included age, BMI, certified laparoscopic technologist, IVPCA with or without fentanyl, and local anesthesia of the wound.

In this study, the anesthesiologist decided on a case-by-case basis for the postoperative use of IVPCA, as no postoperative analgesia protocol was established in advance. However, since IVPCA may affect the frequency of postoperative analgesic use [6], we added an analysis stratified by the presence or absence of IVPCA. Therefore, we divided the patients into subgroups according to the presence or absence of IVPCA and performed the analysis using the same adjustment variables as in the main analysis. In addition, to confirm the robustness of the main analysis, we performed sensitivity analyses with different definitions of the outcome. In the first sensitivity analysis, we defined outcome as the use of acetaminophen, NSAIDs, or IVPCA flushes. We evaluated the association between the procedure and outcome using multivariable logistic regression analysis. A second sensitivity analysis was conducted to evaluate the association between the procedure and outcome using multivariable logistic regression analysis, with outcomes defined as the use of acetaminophen, NSAIDs, or IVPCA flushes, stratified by the presence or absence of IVPCA.

Since this was a small, single-center study, and all cases within the relevant period were included, the sample size based on effect estimation was not calculated. Statistical significance was set at *p* < 0.05. All statistical analyses were performed using EZR (Saitama Medical Center, Jichi Medical University, Saitama, Japan), a graphical user interface for R (The R Foundation for Statistical Computing, Vienna, Austria). More precisely, it is a modified version of the R commander designed to add statistical functions frequently used in biostatistics [7].

## Results

### Study participants

During the study period, 127 patients underwent robotic or laparoscopic total hysterectomies, oh which 3 were excluded and 124 were included in the study (Fig. 1). Robotic hysterectomy was performed in 38 patients, and laparoscopic hysterectomy was performed in 86 patients. The median age was 46.0 years (IQR 44.0–52.0 years), median BMI was 22.0 (IQR 20.2–25.4), 52 (42.0%) of the procedures were performed by laparoscopic technicians, 25 (65.8%) were performed by laparoscopic hysterectomy specialists, IVPCA was used in 42 (33.9%) robotic hysterectomies and 21 (55.3%) laparoscopic hysterectomies, and local anesthesia was used in 22 (17.7%) robotic hysterectomies and 14 (36.8%) laparoscopic hysterectomies (Table 1).

**Table 1:** Preoperative and intraoperative patient characteristics

Variables	Overall (*n* = 124)	Robot (*n* = 38)	Laparoscopy (*n* = 86)
Age (year), median (IQR)	46.0 (44.0, 52.0)	51.0 (43.5, 61.2)	46.0 (44.0, 49.0)
BMI (kg/m^2^), median (IQR)	22.0 (20.2, 25.4)	24.5 (20.3, 27.6)	21.6 (20.2, 23.6)
Anesthesia			
Inhalation anesthesia, *n* (%)	113 (91.1)	35 (92.1)	78 (90.7)
TIVA, n (%)	11 (8.80)	3 (7.90)	8 (9.30)
Fentanyl, mcg, median (IQR)	250 (200, 300)	250 (200, 300)	250 (200, 300)
Operation			
License, *n* (%)	52 (42.0)	25 (65.8)	27 (31.4)
Uterus, g, median (IQR)	194 (121, 282)	125 (81.5, 214)	219 (143, 295)
Surgery time, min, median (IQR)	221 (173, 274)	257 (233, 314)	204 (155, 258)
Postoperative analgesia			
IVPCA, *n* (%)	42 (33.9)	21 (55.3)	21 (24.4)
TAP, *n* (%)	22 (17.7)	14 (36.8)	8 (9.30)

*BMI* body mass index, *TIVA* intravenous patient-controlled analgesia, *IVPCA* patient-controlled analgesia, *TAP* transversus abdominis plane block, *IQR* interquartile range

### Outcomes

The primary outcome of acetaminophen or NSAID use was 10 (26.3%) in the robotic hysterectomy group and 52 (60.5%) in the laparoscopic hysterectomy group. In the IVPCA group, analgesic use was observed in 2 (5.26%) patients in the robotic hysterectomy group and 6 (7.00%) in the laparoscopic hysterectomy group (Table 2).

**Table 2:** Association between outcome and exposure

	Robotic (*n* = 38)	Laparoscopic (*n* = 86)	Unadjusted		Adjusted	
	Use of analgesia, *n* (%)		OR [95% CI]	*p* value	OR [95% CI]	*p* value
Primary analysis	10 (26.3%)	52 (60.5%)	4.28 (1.85–9.93)	< 0.01	2.62 (0.91–7.56)	0.07
Subgroup analyses						
IVPCA-no use	8 (21.1%)	46 (53.5%)	2.72 (0.91–8.12)	0.07	2.20 (0.60–8.12)	0.24
IVPCA use	2 (5.26%)	6 (7.00%)	3.80 (0.67–21.6)	0.13	12.3 (0.93–161)	0.06
Sensitivity analyses 1	13 (34.2%)	58 (67.4%)	3.98 (1.78–8.94)	< 0.01	2.31 (0.87–6.14)	0.09
Sensitivity analyses 2						
IVPCA-no use	8 (21.1%)	46 (53.5%)	2.72 (0.91–8.12)	0.07	2.20 (0.60–8.12)	0.24
IVPCA use	5 (13.2%)	12 (14.0%)	4.27 (1.13–16.1)	0.03	7.94 (0.76–82.6)	0.08

Primary analyses: outcome was the use of acetaminophen, NSAIDs

Sensitivity analyses 1: outcome was the use of acetaminophen, NSAIDs, or IVPCA flushes

Sensitivity analysis 2: subgroup analysis (IVPCA use or not) of sensitivity analysis 1

*IVPCA* intravenous patient-controlled analgesia

### Association between surgical procedure and outcome

In the primary analysis, unadjusted logistic regression analysis showed that laparoscopic surgery was associated with significantly greater use of postoperative analgesia than robotic total hysterectomy (odds ratio [OR] 4.28; 95% confidence interval [CI] 1.85–9.93; *p* < 0.01). However, adjusted logistic regression analysis did not detect a significant difference (OR 2.62; 95% CI 0.91–7.56; *p* = 0.07). In all subgroup and sensitivity analyses, no significant differences were detected in the multivariable logistic regression analysis (Table 2).

## Discussion

### Summary of key findings

Our findings showed no significant difference in the use of postoperative analgesics between robotic and laparoscopic total hysterectomy. The effect of IVPCA was additionally examined in subgroup and sensitivity analyses, with no difference in results.

### Relationship with previous literature

Univariate analysis showed that analgesic use was higher for laparoscopic total hysterectomy than for robotic total hysterectomy. Meanwhile, multivariable analysis showed no significant difference was detected. However, there was a trend toward greater analgesic use with total laparoscopic hysterectomy, with an OR of 2.62 and a 95% CI of 0.91–7.56.

One reason is that robotic surgery may result in less postoperative pain due to using more stable trocars. Previous reports have suggested that robotic surgery, where the trocar is stable at the abdominal wall, may cause less postoperative pain than laparoscopic surgery as trauma to the abdominal wall is reflected in postoperative pain [8, 9]. Also, since this study had a small sample size, it may be possible to detect differences in the percentage of postoperative analgesic use between robotic and laparoscopic total hysterectomy by studying a larger sample size. These points may explain the trend toward greater postoperative analgesic use in the laparoscopic surgery group in the multivariable analysis.

### Implication

This study showed no significant difference in postoperative pain between robot-assisted and laparoscopic total hysterectomy when non-opioid pain management involving NSAIDs and acetaminophen was used. Since robotic surgery is more expensive than laparoscopic surgery [9], it is reasonable to select laparoscopic surgery as an option for minimally invasive total hysterectomy. In addition, a new postoperative pain assessment item, postoperative analgesics, was used in this study, and patients did not require analgesics even when the numerical rating scale (NRS) score was 4–6, suggesting that the interpretation of the NRS may differ between providers and patients [10]. This study is new in the evaluation of postoperative pain as it uses an assessment of whether the patient wants analgesics.

### Limitations

This study has several limitations. First, the sample size is small. Although six variables were adjusted for in the multivariable analysis, the event power variable was < 10. The results might have differed if the sample size had been sufficient. Another limitation was the presence of confounding factors. We adjusted for age, BMI, surgeon skill (certified laparoscopic technologist or not), IVPCA with or without fentanyl, and local wound anesthesia. However, we could not adjust the surgeon’s preference for robotic or laparoscopic total hysterectomy. Further, the outcome (percentage of postoperative analgesia used) did not include opioids.

There are currently no reported outcomes for which opioid and non-opioid use can be evaluated as a single measure. Therefore, opioids were analyzed as adjustment variables in this study. We believe treating only non-opioids as an outcome is reasonable in Japan, where opioids are not frequently used postoperatively. In addition, we used wound local anesthesia instead of the transversus abdominis plane (TAP) block. It has been reported that the TAP block is superior to wound local anesthesia at the port site for postoperative pain in patients undergoing robotic and laparoscopic total hysterectomy [11]. Therefore, using a TAP block instead of wound local anesthesia might have affected the results.

## Conclusion

When multimodal postoperative pain management with NSAIDs and acetaminophen was used, no significant reduction in postoperative pain was detected with robotic total hysterectomy compared with laparoscopic total hysterectomy. Future studies with larger sample sizes and more comprehensive set of intraoperative and postoperative analgesic management protocols remain warranted.

## Data Availability

The datasets generated and analyzed during the current study are available from the corresponding author upon reasonable request.
